# Genetic Variation in *Rheum palmatum* and *Rheum tanguticum* (Polygonaceae), Two Medicinally and Endemic Species in China Using ISSR Markers

**DOI:** 10.1371/journal.pone.0051667

**Published:** 2012-12-18

**Authors:** Xumei Wang, Rui Yang, Shifang Feng, Xiaoqi Hou, Yuqu Zhang, Yan Li, Yi Ren

**Affiliations:** 1 College of Medicine, Xi'an Jiaotong University, Xi'an, China; 2 College of Life Sciences, Shaanxi Normal University, Xi'an, China; Lund University, Sweden

## Abstract

**Aims:**

Both *Rheum palmatum* and *R. tanguticum* are important but endangered medicinal plants endemic to China. In this study, we aimed to (i) investigate the level and pattern of genetic variability within/among populations of those species; (ii) evaluate genetic differentiation between both species and its relationships and ascertain whether both species are consistent with their current taxonomical treatment as separate species; and (iii) discuss the implications for the effective conservation of two species.

**Methods:**

Total 574 individuals from 30 populations of *R. palmatum* and *R. tanguticum* were collected, covering the entire distribution range of two species in China. The genetic variation within and among 30 populations was evaluated using inter-simple sequence repeat (ISSR) markers.

**Important Findings:**

Twelve selected ISSR primers generated a total of 175 fragments, 173 (98.86%) of which were polymorphic. The Nei's gene diversity (*H*) and Shannon's index (*I*) of both species were high at species level (*H* = 0.3107, *I* = 0.4677 for *R. palmatum*; *H* = 0.2848, *I* = 0.4333 for *R. tanguticum*). But for both species, the genetic diversity was low at population level, and average within-population diversity of *R. palmatum* was *H* = 0.1438, *I* = 0.2151, and that of *R. tanguticum* was *H* = 0.1415, *I* = 0.2126. The hierarchical AMOVA revealed high levels of among-population genetic differentiation in both species, in line with the gene differentiation coefficient and the limited among-population gene flow (*R. palmatum*: *Φ_st_* = 0.592, *G_st_* = 0.537, *N_m_* = 0.432; *R. tanguticum*: *Φ_st_* = 0.567, *G_st_* = 0.497, *N_m_* = 0.507). By contrast, only 6.52% of the total genetic variance was partitioned between *R. palmatum* and *R. tanguticum*. Bayesian analysis, UPGMA cluster analysis, and PCoA analysis all demonstrated the similar results. A significant isolation-by-distance pattern was revealed in *R. palmatum* (*r* = 0.547, *P* = 0.010), but not in *R. tanguticum* (*r* = 0.241, *P* = 0.100). Based on these results, effective conservation strategies were proposed for these two species. The small molecular variance between *R. palmatum* and *R. tanguticum* revealed that they had a common ancestor, and we considered that these two species might not be good species.

## Introduction

Preserving genetic diversity of species is one of the primary goals of conservation planning, because long-term survival and evolution of species depend on the maintenance of sufficient genetic variability within and/or among populations to accommodate new selection pressures brought about by environmental changes [1]. Therefore, conservation for endangered or threatened and endemic species should be paid more considerable attention and efforts, as the formulation of effective conservation strategies can only be addressed by detailed population genetic analyses [2].

*Rheum* Section *Palmata* A. Los. (Polygonaceae) is endemic to China, and comprises four described species (*R. officinale* Baill., *R. palmatum* L., *R. tanguticum* (Maxim. ex Regel) Maxim. ex Balf., including var. *tanguticum* and var. *liupanshanense* C. Y. Cheng et T. C. Kao, and *R. laciniatum* Prain) [3]. The former three ones are the original plants of official rhubarb which is a widely used and one of the very famous traditional Chinese medicines as a purgative and anti-inflammatory agent [4]. In Chinese medicinal material markets, dried roots and rhizomes of *R. officinale* are called “south rhubarb”, while that of *R. tanguticum* and *R. palmatum* are called “north rhubarb”.Because the rhubarb from *R. tanguticum* has the best quality, *R. tanguticum* has become endangered and was listed in the China higher plants endangered list due to the overexploitation and the limited distribution [5], [6]. *R. officinale* and *R. palmatum* face great pressure exacerbated by the reduction of the wild resources of *R. tanguticum*, and both are treated as “threatened” species in China. In previous work, we have investigated the genetic diversity of *R. officinale* [7]. Genetic diversity of *R. tanguticum* were reported but based on very limited samples (only collected from Qinghai-Tibet Plateau [5] or from sole Qinghai province [6]). Therefore, the genetic information of *R. tanguticum* was neither been really estimated nor sufficient for the conservation of the endangered but valuable species, and the genetic information for *R. palmatum* remains unknown.

Although the species in Sect. *Palmata* can be easily distinguished from those in other sections by the palmate lobed leaves, the differences among species of Sect. *Palmata* are ambiguous and mainly based on the depth of leaf division, i. e., the leaves of *R. officinale* are lobed, and that of *R. palmatum* are half-parted, whereas, that of *R. tanguticum* and *R. laciniatum* are parted and linear, respectively [3]. It is well-known that morphological characters are prone to environmental influences and may vary during different developmental stages of plants. During our field survey, *R. palmatum* and *R. tanguticum* were found more difficult to be distinguished than *R. palmatum* and *R. officinale*. Many intermediate characters between parted and half-parted leaves can be observed with the increase of populations and/or individuals. In fact, *R. tanguticum* is initially published by Regel as a variety of *R. palmatum* [8]. It should be noted that although *R. laciniatum* is distributed in north of Sichuan [3], any individuals of *R. laciniatum* could not be exactly identified in our field survey according to the morphological characters. The molecular systematic analyses based on very limited samples from different markers are different [5], [9]–[12], but it seems that the relationship between *R. tanguticum* and *R. palmatum* are closer than that of *R. tanguticum* and *R. officinale* [13]. On this basis, we presumed that these two species likely had a common ancestor. *R. palmatum* is mainly distributed in Hebei, Shanxi, Shaanxi, Gansu, Sichuan, Qinghai, and Tibet provinces, while *R. tanguticum* narrowly inhabits in Ningxia, Gansu, Sichuan, and Qinghai provinces. The distribution of those two species is overlapped in northwest China [14]. The two species can be found in the forest edge of hills, in shrubs or in the valleys near rivers.

Among various molecular markers, the inter-simple sequence repeats (ISSR) based on PCR technique, have a better reproducibility than randomly amplified polymorphic DNA (RAPD) [15], [16] and are of easier detection than amplified fragment length polymorphisms (AFLP) and restricted fragment length polymorphisms (RFLP) [17], [18]. Therefore, ISSR has been described as a powerful technique to assess genetic diversity among closely related species and to detect similarities and genetic relationships among and within species [19]–[23].

In the present study, ISSR markers were employed to (1) investigate the level and pattern of genetic variability within/among populations of those species; (2) evaluate genetic differentiation between both species and its relationships and ascertain whether both species are consistent with their current taxonomical treatment as separate species; and (3) discuss the implications for the effective conservation of two species.

## Results

### Genetic diversity

**Figure 1:**
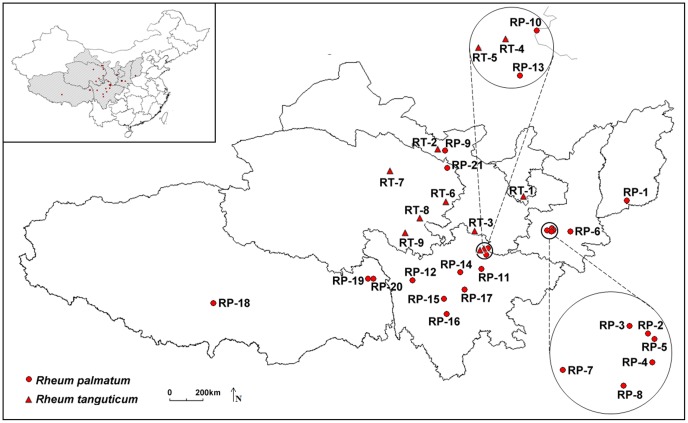
Geographic distribution of the 30 studied populations of *R. palmatum* and *R. tanguticum* in China. For population codes, see Table 1.

Total 574 individuals from 30 populations surveyed across *R. palmatum* and *R. tanguticum* (Table 1, Fig. 1) generated a total of 175 fragments by using 12 selected ISSR primers, of which 173 (98.86%) were polymorphic (Table 2). Each primer amplified from 10 to19 with an average of 14.6. The fragment sizes ranged from 200 to 2000 bp.The size of the amplified fragments ranged from 200 to 2000 bp. Most of fragments were shared across species, and only 5 and 3 bands were unique to *R. palmatum* and *R. tanguticum*, respectively.

**Table 1:** Sampling details of the populations of *R. palmatum* (21) and *R. tanguticum* (9) in the present study.

Species	Population	Locality	Longitude (E)	Latitude (N)	Altitude (m)	Sample size	Voucher
*R. palmatum*	RP1	Zhongcun, Qinshui county, Shanxi (Mts. Zhongtiao)	111°57.259′	35°25.133′	1766	20	Xiao-Qi Hou 10062901
	RP2	Wengongmiao, Mei county, Shaanxi (Mts. Qinling)	107°46.810′	33°58.691′	3423	20	Xiao-Qi Hou 10072209
	RP3	Mingxingsi, Mei county, Shaanxi (Mts. Qinling)	107°43.898′	33°59.796′	2859	20	Xiao-Qi Hou 10072105
	RP4	Nantianmen, Mei county, Shaanxi (Mts. Qinling)	107°47.400′	33°55.284′	2652	20	Xiao-Qi Hou 10072413
	RP5	Xiabansi, Mei county, Shaanxi (Mts. Qinling)	107°47.649′	33°58.176′	2599	20	Xu-Mei Wang 10061001
	RP6	Fengyu, Hu county, Shaanxi (Mts. Qinling)	108°45.600′	33°52.200′	2578	20	Xu-Mei Wang 2010071401
	RP7	Huangbaiyuan, Taibai county, Shaanxi (Mts. Qinling)	107°33.479′	33°53.968′	2400	20	Yu-Qu Zhang 9081003
	RP8	Longdonggou, Zhouzhi county, Shaanxi (Mts. Qinling)	107°42.816′	33°51.982′	2658	18	Yu-Qu Zhang 09080801
	RP9	Wangbalangyan, Yongchang county, Gansu (Mts. Qilian)	101°51.731′	38°06.303′	3006	20	Xu-Mei Wang 08071807
	RP10	Tielou, Wen county, Gansu (Mts. Qinling)	104°17.227′	32°55.593′	3234	19	Yu-Qu Zhang 10091219
	RP11	Baodinggou, Mao county, Sichuan (Mts. Min)	103°54.924′	31°55.920′	3102	15	Yu-Qu Zhang, Xiao-Qi Hou 09083001
	RP12	Dagai, Xinlong county, Sichuan (Mts. Daxue)	100°03.080′	31°17.926′	3760	20	Yi Ren 9082516
	RP13	Xiaohe, Songpan county, Sichuan (Mts. Min)	104°09.745′	32°36.110′	2749	20	Yu-Qu Zhang, Xiao-Qi Hou 09090607
	RP14	Miyaluo, Li county, Sichuan (Mts. Qionglai)	102°45.440′	31°46.328′	3579	20	Yu-Qu Zhang 10081809
	RP15	Yala, Kangding county, Sichuan (Mts. Daxue)	101°51.833′	30°14.782′	3692	20	Yu-Qu Zhang 1008090218
	RP16	Moxi, Luding county, Sichuan (Mts. Daxue)	101°59.003′	29°34.177′	3204	20	Yu-Qu Zhang 10082412
	RP17	Wolong, Wenchuan county, Sichuan (Mts. Qionglai)	102°57.942′	30°53.048′	3590	20	Yu-Qu Zhang 10081305
	RP18	Rendui, Nanmulin county, Tibet (Mts. Gangdisi)	89°05.502′	30°07.875′	4498	20	Yi Ren 9081708
	RP19	Tuoba, Changdu county, Tibet (Mts. Daxue)	97°41.455′	31°21.284′	4418	20	Yi Ren 09082111
	RP20	Qingnidong, jiangda county, Tibet (Mts. Daxue)	97°54.333′	31°22.567′	4000	18	Yi Ren 9082114
	RP21	Xianmi, Menyuan county, Qinghai (Mts. Qilian)	101°59.934′	37°11.549′	3148	18	Xiao-Qi Hou 10081546
*R. tanguticum*							
var. *liupanshanense*	RT1	Longwangmiaogou, Jingyuan county, Ningxia (Mts. Liupan)	106°13.003′	35°40.110′	2224	20	Xu-Mei Wang 08072513
var. *tanguticum*	RT2	Heilingou, Yongchang county, Gansu (Mts. Qilian)	101°26.100′	38°10.002′	2575	20	Xu-Mei Wang 08071910
	RT3	Axiagou, Diebu county, Gansu (Mts. Min)	103°31.498′	33°48.152′	4000	7	Xi-Chun Du ax1533
	RT4	Wanglang, Pingwu county, Sichuan (Mts. Min)	104°03.010′	32°52.434′	3193	19	Yu-Qu Zhang, Xiao-Qi Hou 09091121
	RT5	Huanglong, Songpan county, Sichuan (Mts. Min)	103°52.469′	32°47.864′	3597	20	Yu-Qu Zhang, Xiao-Qi Hou 09090812
	RT6	Maixiu, Zeku county, Qinghai (Qinghai-Tibet Plateau)	101°55.872′	35°18.872′	3349	20	Xiao-Qi Hou 10080905
	RT7	Xinyuan, Tianjun county, Qinghai (Qinghai-Tibet Plateau)	98°51.398′	37°05.61′	3693	20	Xiao-Qi Hou 10081223
	RT8	Jungong, Maqin county, Qinghai (Qinghai-Tibet Plateau)	100°33.970′	34°36.955′	3373	20	Xiao-Qi Hou 10081332
	RT9	Jimai, Dari county, Qinghai (Qinghai-Tibet Plateau)	99°42.669′	33°49.151′	3947	20	Xiao-Qi Hou 10081435

**Table 2:** ISSR primers used for ISSR analysis in the present study.

Primer code	Sequence (5′→3′)	Annealing temperature (°C)	No. of amplified bands	No. of polymorphic bands
UBC807	(AG)_8_T	51	12	12
UBC811	(GA)_8_C	53	13	13
UBC816	(CA)_8_T	52	16	15
UBC825	(AC)_8_T	52	12	12
UBC835	(AG)_8_YC	52	10	10
UBC836	(AG)_8_YA	52	16	16
UBC841	(GA)_8_YC	52	15	14
UBC842	(GA)_8_YG	56	11	11
UBC888	BDB(CA)_7_	52	19	19
UBC889	DBD(AC)_7_	52	17	17
UBC890	VHV(GT)_7_	56	18	18
UBC891	HVH(TG)_7_	52	16	16

Y = (C, T); B = (C, G, T); D = (A, G, T); H = (A, G, T); V = (A, C, G).

In general, ISSR variation within populations was very low in each species, and varied erratically across localities (Table 3). In *R. palmatum*, the highest and the lowest genetic diversities were observed in populations RP12 (Nei's gene diversity *H* = 0.1858, Shannon information index *I* = 0.2742) and RP13 (*H* = 0.1114, *I* = 0.1675), respectively. In *R. tanguticum*, the highest genetic diversity existed in population RT1 (*H* = 0.1678, *I* = 0.2495) and the lowest in RT6 (*H* = 0.1133, *I* = 0.1675). The average within-population diversity of *R. palmatum* (*H* = 0.1438, *I* = 0.2151) was slightly higher than that of *R. tanguticum* (*H* = 0.1415, *I* = 0.2126), and the former also harbored more ISSR diversity at the species level (*H* = 0.3107 vs. 0.2848, *I* = 0.4677 vs. 0.4333).

**Table 3:** Genetic diversity within the populations of *R. palmatum* and *R. tanguticum*.

Species	Populations	*N_a_*	*N_e_*	*H*	*I*	*PPB* (%)
*R. palmatum*	RP1	1.5086	1.2884	0.1716	0.2584	50.86
	RP2	1.3657	1.1991	0.1187	0.1799	36.57
	RP3	1.3543	1.2008	0.1180	0.1775	35.43
	RP4	1.4343	1.2626	0.1529	0.2284	43.43
	RP5	1.3714	1.2067	0.1216	0.1836	37.14
	RP6	1.4800	1.2681	0.1596	0.2411	48.00
	RP7	1.4971	1.2760	0.1635	0.2469	49.71
	RP8	1.4800	1.2891	0.1701	0.2546	48.00
	RP9	1.3714	1.2187	0.1290	0.1935	37.14
	RP10	1.4686	1.2813	0.1632	0.2432	46.86
	RP11	1.3543	1.2224	0.1305	0.1937	35.43
	RP12	1.5029	1.3253	0.1858	0.2742	50.29
	RP13	1.3314	1.1883	0.1114	0.1675	33.14
	RP14	1.3829	1.2337	0.1359	0.2026	38.29
	RP15	1.4629	1.2527	0.1501	0.2273	46.29
	RP16	1.4171	1.2496	0.1438	0.2145	41.71
	RP17	1.4686	1.2957	0.1705	0.2529	46.86
	RP18	1.3371	1.2103	0.1233	0.1834	33.71
	RP19	1.3829	1.2438	0.1406	0.2083	38.29
	RP20	1.3314	1.2111	0.1223	0.1814	33.14
	RP21	1.3943	1.2364	0.1370	0.2042	39.43
	Average	1.4142	1.2457	0.1438	0.2151	41.42
	Species level	1.9829	1.5294	0.3107	0.4677	98.29
*R. tanguticum*						
var. *liupanshanense*	RT1	1.4800	1.2910	0.1678	0.2495	48.00
var. *tanguticum*	RT2	1.4514	1.2781	0.1619	0.2412	45.14
	RT3	1.4457	1.2422	0.1426	0.2168	44.57
	RT4	1.4000	1.2312	0.1356	0.2041	40.00
	RT5	1.4057	1.2056	0.1232	0.1882	40.57
	RT6	1.3086	1.1991	0.1133	0.1675	30.86
	RT7	1.3714	1.2020	0.1223	0.1859	37.14
	RT8	1.4171	1.2498	0.1444	0.2154	41.71
	RT9	1.4914	1.2772	0.1627	0.2447	49.14
	Average	1.4190	1.2418	0.1415	0.2126	41.90
	Species level	1.9143	1.4767	0.2848	0.4333	91.43

*N_a_*: observed number of alleles; *N_e_*: effective number of alleles; *H*: Nei's (1973) gene diversity; *I*: Shannon's information index; *PPB*: percentage of polymorphic bands.

### Genetic differentiation and gene flow

The hierarchical AMOVA for all ISSR data set showed that only 6.52% of total genetic variance was partitioned between *R. palmatum* and *R. tanguticum*, while most of this variation still resided among populations within species (93.48%) (Table 4). By contrast, pronounced levels of among-population genetic differentiation were detected within each species as well as the limited among-population gene flow (*R. palmatum*: *Φ_st_* = 0.592, Nei's genetic differentiation index among populations *G_st_* = 0.537, gene flow *N_m_* = 0.432; *R. tanguticum*: *Φ_st_* = 0.567, *G_st_* = 0.497, *N_m_* = 0.507).

**Table 4:** Analysis of molecular variance (AMOVA) in all 30 populations of *R. palmatum* (21) and *R. tanguticum* (9) using 12 ISSR markers.

Source of variation	d.f.	SS	VC	TVP (%)	*P*-value*
*R. palmatum*+*R. tanguticum*					
Between species	1	516.077	2.06166	6.52	<0.001
Within species	572	16904.529	29.55337	93.48	<0.001
*R. palmatum*					
Among populations	20	7458.089	18.53946	59.19	<0.001
Within populations	387	4945.837	12.77994	40.81	<0.001
*R. tanguticum*					
Among populations	8	2523.058	16.51608	56.73	<0.001
Within populations	157	1977.544	12.59582	43.27	<0.001

d.f., degree of freedom; SS, sum of squares; VC, variance components; TVP, total variance percentage;

* Significance tests after 1000 permutations.

On some mountains, more than one population was sampled. For instance, there were eight populations of *R. palmatum* in Mts. Qinling and five populations of *R. palmatum* in Mts. Daxue, and three populations of *R. tanguticum* in Mts. Min**.** To investigate the gene flow among populations in the same region, both *Gst* and *Nm* were analyzed for eight Mts. Qinling populations (RP2, RP 3, RP 4, RP 5, RP 6, RP7, RP8 and RP10), five Mts. Daxue populations (RP12, RP15, RP16, RP19 and RP20) and three Mts. Min populations (RT3, RT4 and RT5). Both *Gst* and *Nm* was 0.470 and 0.563 for eight populations from Mts. Qinling, 0.436 and 0.646 for five populations from Mts. Daxue, and 0.351 and 0.927 for three populations from Mts. Min.,

### Genetic relationships

POPGENE analysis revealed that Nei's unbiased genetic distances ranged from 0.0992 (RP19 vs. RP20) to 0.3324 (RP3 vs. RP21) in *R. palmatum*, and from 0.1015 (RT3 vs. RT5) to 0.3165 (RT1 vs. RT6) in *R. tanguticum*.

**Figure 2:**
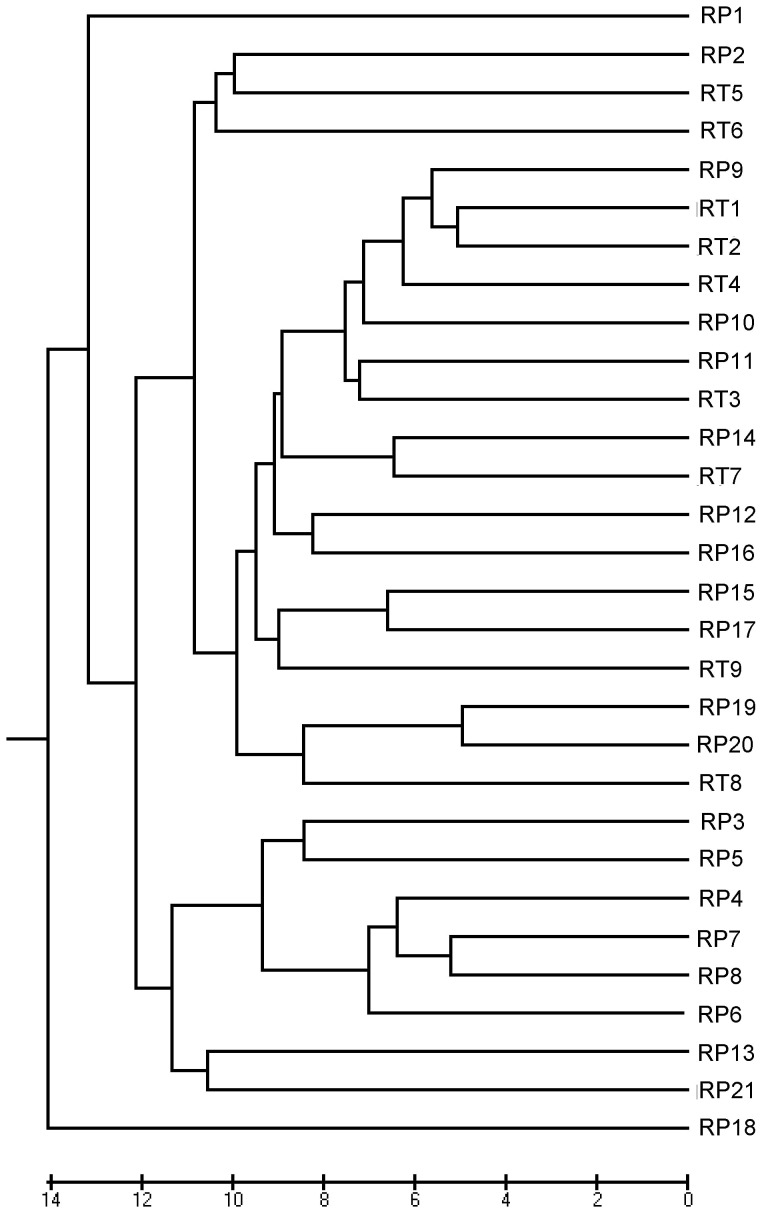
UPGMA dendrogram based on Nei's (1978) genetic distances among the populations of *R. palmatum* and *R. tanguticum*.

The UPGMA tree based on Nei's unbiased genetic distance clustered all populations into two major groups (Fig. 2). Population RP18 in *R. palmatum* from Nanmulin county of Tibet formed a sole group, and the remaining populations of *R. palmatum* and *R. tanguticum* formed the other group, which can be divided into two subgroups. One subgroup included RP1 population in *R. palmatum* from Qinshui county of Shanxi province, and the other contained populations from RP2 to RP17 (16 populations), from RP19 to RP21 (3 populations), and all populations in *R. tanguticum*. The populations from a same species were not clustered in the same group or subgroup.

**Figure 3:**
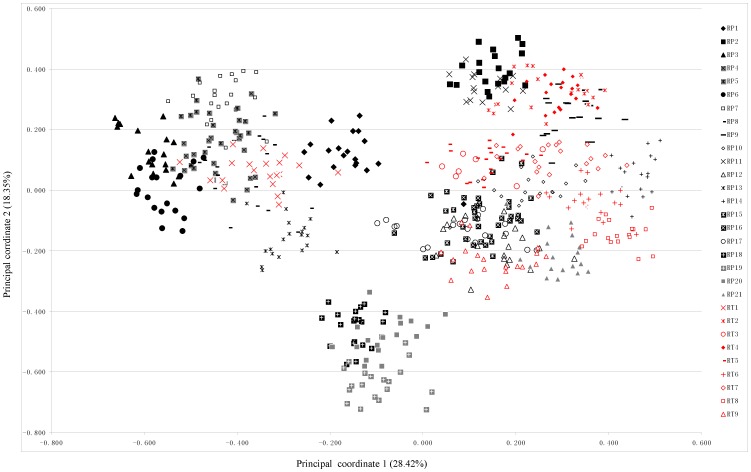
Principal Coordinates Analysis (PCoA) using ISSR data of 574 individuals of *R. palmatum* and *R. tanguticum*.

The two-dimensional PCoA analysis for all 574 individuals in both species accounted for 28.42% (axis 1) and 18.35% (axis 2) of total variance, respectively (Fig. 3). In the PCoA-plot, those populations in *R. palmatum* (RP1 from Shanxi, all populations from Shaanxi except RP2) and the only population from *R. tanguticum* var. *liupanshanense* (RT1 from Ningxia) occupied similar position along the axis 1. Three populations from Tibet (RP18, RP19 and RP20) in *R. palmatum* also had similar genetic similarity. These results showed that both *R. palmatum* and *R. tanguticum* formed a conspicuous close-knit group.

**Figure 4:**
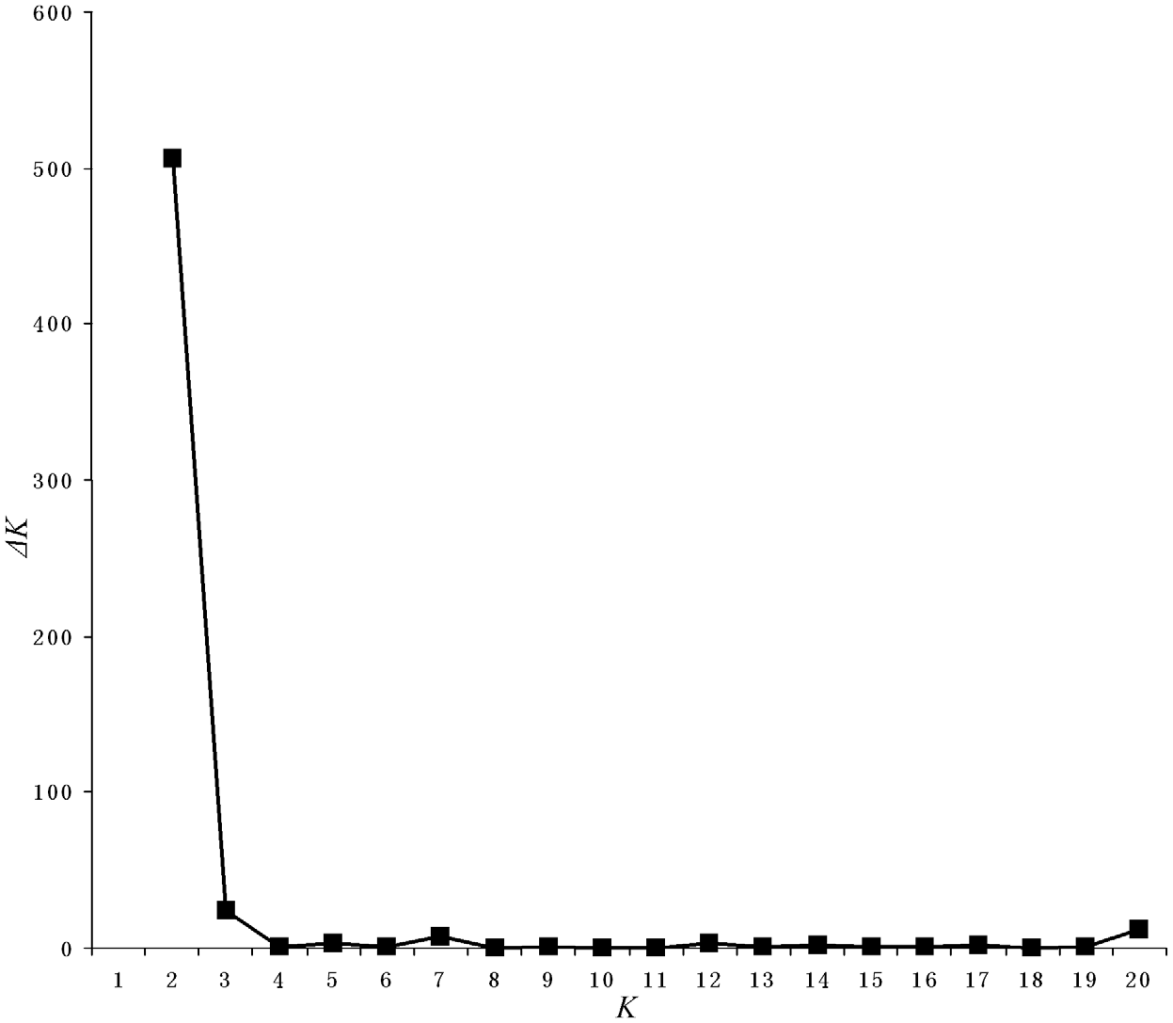
Results of the Bayesian assignment analysis using the program STRUCTURE. The *ΔK* (Evanno et al. 2005) was plotted against various values of *K*, suggesting *K* = 2 as the most likely number of clusters.

**Figure 5:**
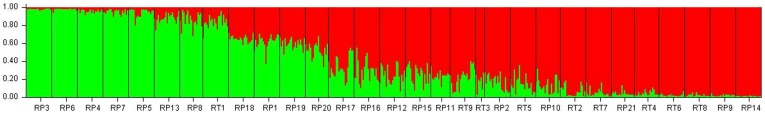
Genetic relationships among the 30 populations of *R. palmatum* and *R. tanguticum* (574 individuals) estimated using STRUCTURE program based on ISSR data. The model with *K* = 2 showed the highest Δ*K* value.

In the ISSR admixture analysis using STRUCTURE, the real *K* value with the highest value of Δ*K* for the 574 individuals was *K* = 2 (Fig. 4). The proportions of each individual in each population assigned into two clusters (cluster I and cluster II) (Fig. 5) which result is in agreement with UPGMA dendrogram. However, some populations (e.g. RP1, RP11, Rp12, RP15, RP16, RP17, RP18, RP19, and RP20) displayed some degree of mixed ancestry though they were identified as *R. palmatum* based on morphological characteristics, and this situation also occurred in *R. tanguticum* populations (e.g. RT3, RT5 and RT9).

The Mantel's test results showed that genetic distances among populations in *R. palmatum* were positively correlated with their geographical distances with significance (*r* = 0.547, *P* = 0.010, 999 permutations), indicating a significant isolation-by-distance pattern. However, no significant correlation between genetic distances and geographical distances was found for populations in *R. tanguticum* (*r* = 0.241, *P* = 0.100, 999 permutations).

## Discussion

### Genetic diversity

Genetic diversity is one of the most important attributes to any population. Environments are constantly changing, and genetic diversity is necessary if populations are to continuously evolve and to adapt to new situations [24]. Therefore, an assessment of genetic diversity is crucial for management and developing effective conservation strategies for a species, especially for the endemic and endangered species. Our results showed that genetic diversities were pretty low at population level (*R. palmatum*: *PPB* = 41.42%, *H* = 0.1438, *I* = 0.2151; *R. tanguticum*: *PPB* = 41.90%, *H* = 0.1415, *I* = 0.2126), and relatively higher at species level (*R. palmatum*: *PPB* = 98.29%, *H* = 0.3107, *I* = 0.4677; *R. tanguticum*: *PPB* = 91.43%, *H* = 0.2848, *I* = 0.4333), which is similar to the level of genetic diversity reported by Hu et al. on *P. tanguticum* (*PPB* = 92.94%, *H* = 0.2689 *I* = 0.4163) [6]. The genetic diversity of *R. palmatum* was higher than that of *R. tanguticum*, but it was slightly lower than that of *R. officinale* (*PPB* = 95.24%, *H* = 0.3341, *I* = 0.5000 [7]). Although the *PPB* of *R. palmatum* was the highest among three species, *PPB* is just a rough estimate of genetic polymorphism due to its sensitivity to sample sizes, and less reliable than Nei's gene diversity index or Shannon's information index [24]. Overall, either *R. palmatum* or *R. tanguticum* maintained a higher genetic diversity at the species level when compared to other long-lived perennial herbs (*H* = 0.1240, *PPB* = 39.30%) based on isozyme analysis [25] and other species in Polygonaceae [26], [27]. The present study also confirmed that widely distributed species (i.e. *R. palmatum*) usually have higher intraspecific genetic diversity than their narrowly distributed counterparts (i.e. *R. tanguticum*) [28].

The high intraspecific genetic diversity of *R. palmatum* and *R. tanguticum* is probably related to its unique evolutionary history. Fossil records indicated the occurrence of Polygonaceae at least as far as the Paleocene [29] and the divergence of *Rheum* and its sister groups was dated to the Miocene, about 22 million years before present, based on a molecular clock hypothesis [30], [31], hence both species have probably accumulated large quantities of genetic variations. Mating systems have been postulated to be one of the most important factors that determine the genetic diversity in plant species [32]. The species in *Rheum* are anthophilous [33] and self-incompatible, and its trigonous achenes have wings, which help the fruits easily disperse by wind. On the other hand, as long-lived herbaceous plants, both *R. palmatum* and *R. tanguticum* have more opportunity to accumulate mutant. Moreover, the natural geographic distribution may affect the genetic diversity level for a species and the widespread species have higher level of genetic variability than narrowly distributed ones in general [34]. *R. palmatum* and *R. tanguticum* are widespread in China, especially for *R. palmatum* which is distributed from southern Shanxi to the central of Tibet [14]. The genetic diversity was low for both species at population level when comparing with that at species level. The reasons for this may be attributed to: firstly, the populations of two species are isolated in their distribution, as the geographically isolated populations of a species usually have slightly reduced levels of genetic diversity when compared with those populations from areas where the species is abundant and continuously distributed [35]; secondly, because the roots and rhizomes are traditionally used for medicinal purpose and excessive excavation of these herbs has caused a tremendous decrease of individuals in the field, only seven individuals were found in RT3 for example, and it is difficult to find the seedlings in the destroyed populations. Therefore, according to our results, we may concluded that the endangered status of these two species were probably due to over-harvesting of the wild resources, rather than a lack of the genetic diversity.

### Genetic differentiation

High level of among-population genetic differentiation was revealed in both *R. palmatum* (*Φ_ST_* = 0.592, *G_ST_* = 0.537) and *R. tanguticum* (*Φ_ST_* = 0.567, *G_ST_* = 0.497), and both were slightly lower than *R. officinale* (*Φ_ST_* = 0.7438, *G_ST_* = 0.6438) [7]. When compared with the previous reports on *R. tanguticum* revealed by ISSR (*Φ_ST_* = 0.290, *G_ST_* = 0.3585 [6] or SSR markers (*Φ_ST_* = 0.2118, *G_ST_* = 0.249 [5]), both species held a relatively high genetic variation among-populations. Similar results were also found in other endangered or endemic species, such as *Megacodon stylophorus* (Gentianaceae) [36], *Rhodiola alsia* (Crassulaceae) [37], and *Torreya jackii* [38].

The high level of population genetic differentiation within a species may be explained by several factors, including geographic distribution, breeding system, genetic drift or genetic isolation of populations [39]. The scattered distribution of a species and topographic barriers can lead to difficulties in pollen and seed dispersal, and consequently, to limited gene flow among populations [26]. Mantel test showed a significant isolation-by-distance pattern in *R. palmatum*, indicating that geographic isolation has significant effect on genetic structure in this species. Samples of *R. palmatum* in this study covered the whole distribution range. *R. palmatum* growths only in related higher altitude mountains (e.g. Mts. Qionglai, Mts. Qilian, Mts. Min, Mts. Qingling, and Mts. Daxue), and this isolated distribution undoubtedly restricts gene exchange via seed or pollen dispersal. *R. palmatum* and *R. tanguticum* are pollinated by insects [33], therefore, long-distance pollen movement among populations is limited. The UPGMA phenogram also revealed this genetic differentiation caused by geographical isolation, such as populations RP1 and RP18, the two most divergent populations, locate at the most west and the most east of recorded distribution of the species, respectively (Fig. 2). Both species have numerous small flowers in a panicle and can produce a large amount of seeded achenes can be produced from one plant. The seeds are small sized (3–4 mm long and 1–2 mm in diameter), relatively light (ca. 7 g/1000 pcs when dried) [40], and probably dispersed over limited distances by wind because the achenes are encapsulated by winged persistent tepals. On the other hand, the seed germination rate of *R. palmatum* and *R. tanguticum* is 41% and 48% in the field, respectively [40], which may impede successful expansion and therefore reduce effective gene flow among populations. Because of this regional distribution and pollen and seed dispersal pattern, high level of population differentiation was expected. Therefore, the limited gene flow was detected both in *R. palmatum* (*N_m_* = 0.432) and *R. tanguticum* (*N_m_* = 0.507), which enhance the genetic differentiation among populations in each species. Gene flow among populations on the same mountain for both species was also restricted. FThe *Nm* of eight populations in *R. palmatum* from Mts. Qinling and five populations in *R. palmatum* from Mts. Daxue was 0.563 and 0.646, respectively, while the *Nm* of three populations in *R. tanguticum* from Mts. Min was 0.927. Wright determined that *Nm*>1 is sufficient to overcome the effects of genetic drift [41]. Thus, the gene flow among populations in both species were not abundant enough to prevent genetic differentiation caused by genetic drift. Meanwhile, populations with continually small effective population sizes are especially susceptible to the loss and reorganization of variation by genetic drift (e.g. 7 individuals of RT3 from Diebu). The Mantel test further clarified that genetic differentiation does not show any spatial pattern and there is no significant correlation between genetic distance and geographic distance for *R. tanguticum*, which also provides further evidence of the existence of genetic drift [42]. No significant isolation-by-distance pattern was detected in *R. tanguticum*, implying that gene flow might also have occurred among geographically distant populations. This possibility cannot be ruled out because this species has a long history of cultivation. It has been widely cultivated in Qinghai and Gansu provinces, and long-distance inter-regional exchange of seedlings or seeds may have happened due to anthropogenic activities.

### Genetic relationships

In China medicinal material markets, it is well known that the rhubarb derived from roots and rhizomes of *R. palmatum* and *R. tanguticum* is called “north rhubarb”, which has the best quality. However, both species are morphologically similar in many characteristics (e.g. purple-red tepals and leaf shape), and it is very difficult to identify them. According to the description of Flora of China [3], the characteristics often used to identify these species are the depth of leaf blade dissection and shape of the lobes that is usually judged subjectively. In fact, many transitional types between two species were found in overlapped distribution region during our field survey. In recent years, molecular data has been used to revolve the relationship between two species [9]–[13]. However, the relationship of them is still ambiguous, largely due to very small and limited sampling size or poorly informative characters.

In the present study, ISSR makers provided an alternative approach to examine the relationship of both species at DNA level. As it is expected that very low genetic variation (*Φ_ST_* = 0.0652) was detected between *R. palmatum* and *R. tanguticum*, which indicates that these two morphologically closely related species are genetically similar. The samples used in this study were collected from the whole distribution regions of two species, and thus were more representative than those in previous studies [9]–[13] with limited individuals. The UPGMA tree and the PCoA analysis indicated that populations/individuals of *R. palmatum* are nested with that of *R. tanguticum* (Fig. 2, 3). It should be noted that some populations (e.g. RP1, RP11, Rp12, RP15, RP16, RP17, RP18, RP19, RP20) displayed some degree of mixed ancestry thoughthey were identified as *R. palmatum* based on morphological characteristics, and this situation also occurred in *R. tanguticum* populations (e.g. RT3, RT5, RT9) (Fig. 5). In addition, interspecific hybridization may happen in both species, leading to produce a lot of intermediate individuals [5]. On the other hand, we also measured depth of leaf blade dissection and width of the principal lobes of both species, and the results showed that the difference among all individuals was gradational rather than distinct (unpublished data). The results of the present study indicated that the genetic relationships among populations of *R. palmatum* and *R. tanguticum* were not consistent with their current taxonomical treatment as separate species.

### Implications for taxonomy

A new species *R. qinlingense* Y. K. Yang, D. K. Zhang et J. K. Wu in the Sect. *Palmata* was reported, which was collected at Taibai county, Shaanxi province (Mts. Qinling) according to the diverse leaf characteristics, i.e. the depth of leaf division (undulate, lobed, half-parted and parted) [43]. However, Nei's original measures of genetic distance in this study (data not shown) and the UPGMA dendrogram (Fig. 2) failed to provide support for their conclusions. The largest and the smallest genetic distances between RP7 (*R. qinlingense*) and other populations was 0.3068 (RP18) and 0.1043 (RP8), respectively, with a mean of 0.2149. Cluster analysis showed strong correlations between RP7 and RP8, as well as RP4 and RP6. RP7 was not classified as one group based on ISSR markers. SinceRP7 did not stand alone in these analyses, it is necessary to deliberate when classifying it as a separate species.

### Implications for conservation of wild resources

The results of the present study showed that both species have high genetic diversity at species level, however, extraordinarily high among-population genetic differentiation existed in both species. As high genetic differentiation resided among the populations of each species, also each of the remaining populations could represent a large proportion of the genetic variation of these two species; therefore, great efforts should be made to preserve all the extant populations and their habitats in the field, especially for those populations with higher genetic diversity, e.g., RP1 and RP12 in *R. palmatum*, and RT9 in *R. tanguticum*, should be given priority for *in situ* conservation, and their habitats should be protected and the exploitation of wild resources be forbidden. If *ex situ* conservation is required in gardens, samples should be collected from as many populations as possible. Considering both *R. palmatum and R. tanguticum* have a bulk demands and a long history of utilization in China, and their wild resources have long been subject to excessive collection. It would be good if sufficient artificial plantations can be established and meet the market demands for these two species. Only in this way can the excessive collection of their wild resources be alleviated.

## Materials and Methods

### Ethics statement

According to regulations of the People's Republic of China on wild plants protection, the permits are required only for those species included in the list of state-protected plant species when it is collected. Neither *R. palmatum* nor *R. tanguticum* is in the list of state-protected plant species (Yu YF, A milestone of wild plants protection in China - the list of wild plants protected by the nation (the first batch), Plants 1999 (5): 3–11; Regulations of the People's Republic of China on wild plants protection, http://www.people.com.cn/item/faguiku/zrzyf/U1020.html). Thus, no specific permits were required for the described field studies. During the samples collection, only a small fraction of a blade was collected to avoid causing any harm to the plants and their habitats.

### Plant materials

From 2008 to 2010, thirty populations were collected throughout the distribution of both *R. palmatum* (21 populations) and *R. tanguticum* (9 populations, 8 of them are from var. *tanguticum* and one from the only distribution site of var. *liupanshanense*), including Hebei, Shanxi, Shaanxi, Gansu, Sichuan, Qinghai, Ningxia, and Tibet provinces in China. Fresh leaves from 18–20 individuals (only seven individuals in RT3 population from Gansu) were collected randomly in each population, depending on accessibility and population size. Each population was positioned by a GPS, and the detailed locations of the studied populations are listed in Table 1 and Figure 1. The young leaves were stored and dried in ziplock bags with silica gel and transported back to the laboratory for DNA extraction. The voucher specimens were deposited in the Herbarium of Shaanxi Normal University (SANU).

### DNA extraction and PCR amplification

Total DNA was extracted from the silica gel-dried leaves using the modified 2× CTAB procedure [44]. The quality and quantity of DNA were performed by UV-spectrophotometer (ND-2000, NanoDrop, USA). DNA concentration and purity were also determined by electrophoresis on 1.0% agarose gels based on the intensities of band when compared with l kb plus DNA ladder as marker. The DNA samples were diluted to the concentration of 50 ng/µl and stored at −20°C for use.

One hundred ISSR primers synthesized by Sangon Biological Engineering Technology & Service (Shanghai), according to the primer set published by University of British Columbia, Canada (UBC set No. 9) were used for amplification to standardize the PCR conditions. Twelve of one hundred ISSR primers produced clear, reproducible and relatively high polymorphism bands were selected to amplify all 574 samples (Table 2). The PCR amplification was carried out according to our previous study for the optimized ISSR-PCR reactions of *R. officinale* [45]. PCR products were electrophoresed on 1.6% (w/v) agarose gels, in 1× TBE Buffer at 110 V for 1.5 h and stained with ethidium bromide (0.5 µg/ml). Gels with amplification fragments were visualized and photographed in UV light by using Bio-Rad Gel Documentation System (Bio-Rad Laboratories, UK). DL2000 ladder (TaKaRa Biotechnology, China) was used as DNA molecular weight.

### Statistical analysis

The amplified fragments, with the same mobility according to their molecular weight (bp), were scored in terms of a binary code as present (1) or absent (0). Only those consistently reproducible bands were scored, and smeared and weak bands were excluded. The following genetic diversity parameters were calculated for each population and species using POPGENE version 1.32 [46]: the percentage of polymorphic bands (*PPB*), Nei's gene diversity (*H*) [47], Shannon's index (*I*) [48], Nei's unbiased genetic distance [49], Nei's genetic differentiation index among populations (*G_ST_*) [47], and gene flow (*Nm*). An estimate of *Nm* among populations was computed using the formula of *Nm* = 0.5(1−*G_ST_*)/*G_ST_* [50]. The obtained genetic distance matrix was then used to perform the cluster analysis and construct the unweighted pair-group method with arithmetic average (UPGMA) dendrogram using MEGA 4.0 [51]. Principal Coordinates Analysis (PCoA) was also carried out to identify the genetic similarity of the populations using the software package GenAlEx v6.4 [52].

Analyses of molecular variance (AMOVA) were performed on the Euclidean distances among ISSR phenotypes by using ARLEQUIN to deduce the significance of genetic divisions between both species as well as among populations within each species. Geographic distances were interpreted by the latitudes and longitudes with Mapinfo 8.0 Program. Mantel test of genetic and geographic distances was carried out to evaluate the correlation between the two data matrices using TFPGA software [53] (computing 999 permutations).

Finally, to test for genetic admixture across species boundaries (*viz*. hybridization), a Bayesian analysis of ISSR population structure was performed on the entire data set using the program STRUCTURE (version 2.3) [54] to detect population structure and estimate the number of populations (*K*) in a sample and to assign individuals to one or more of these populations (*K*). The number of genetically distinct clusters (*K*) was set to vary from 1 to 21. The model was run for eight independent simulations for each *K*, used a burn-in length of 50,000 and a run length of 100,000 iterations. Other parameters were set to default values. Following the program's dominant marker settings, the “no admixture” model was used, and uncorrelated allele frequencies among populations were assumed. The most likely number of clusters was estimated according to the model value (Δ*K*) based on the second order rate of change, with respect to *K*, of the likelihood function, following the procedure described by Evanno et al. [55].

## Conclusions

In summary, genetic diversity was low at population level but high at species level for both *R. palmatum* and *R. tanguticum* revealed by ISSR markers. High genetic differentiation among populations for both species was attributed to the geographical isolation and genetic drift. The observed genetic structure of populations in both *R. palmatum* and *R. tanguticum* implied that as many populations as possible should be collected when making conservation strategy. The small molecular variance between both species indicated that they had a common ancestor, and these two species might not be good species.
